# Supplementary material for: A systematic review and meta-analysis of knowledge, attitudes, and practices toward anthrax prevention and control in Ethiopia: Implication for a one health policy
Source: PLoS Negl Trop Dis. 2026 Jan 23;20(1):e0013930. doi: 10.1371/journal.pntd.0013930 (PMC12854452; doi:10.1371/journal.pntd.0013930)
Supplement: S2 File — (PDF) [file pntd.0013930.s002.pdf]

Database Search Strings

1. **Pub-Med (MedLine) Database**

(((((((((((((("Anthrax"[Mesh] OR “anthrax” [Title/Abstract] OR "Bacillus anthracis"[Title/Abstract]) AND ("Knowledge, Attitudes, Practice"[Mesh] OR knowledge [Title/Abstract] OR attitude*[Title/Abstract] OR practice*[Title/Abstract] OR KAP [Title/Abstract] OR awareness [Title/Abstract] OR perception*[Title/Abstract] OR belief*[Title/Abstract] OR behaviour*[Title/Abstract] OR behavior*[Title/Abstract] OR "One Health"[title/abstract]) AND ("Ethiopia"[Mesh] OR Ethiopia [Title/Abstract]).

2. **Scopus Database**

TITLE-ABS-KEY (anthrax OR "Bacillus anthracis") AND TITLE-ABS-KEY (knowledge OR attitude*OR practice* OR KAP OR awareness OR perception* OR belief* OR behaviour* OR behavior* OR "one health") AND TITLE-ABS-KEY (Ethiopia)

3. **Web of Science Database**

TS= (Anthrax OR "Bacillus anthracis") AND TS= (knowledge OR attitude* OR practice* OR KAP OR awareness OR perception* OR belief* OR behaviour* OR behavior* OR "one health") AND TS= (Ethiopia)

4. **CAB Abstracts Database**

(Anthrax OR "Bacillus anthracis")AND (knowledge OR attitude* OR practice* OR KAP OR awareness OR perception* OR belief* OR behaviour* OR behavior* OR "one health") AND (Ethiopia)

5. **AGRICOLA Database**

(Anthrax OR "Bacillus anthracis") AND (knowledge OR attitude* OR practice* OR KAP OR awareness OR perception* OR belief* OR behaviour* OR behavior* OR "one health") AND (Ethiopia)
