# Supplementary material for: A systematic review and meta-analysis of knowledge, attitudes, and practices toward anthrax prevention and control in Ethiopia: Implication for a one health policy
Source: PLoS Negl Trop Dis. 2026 Jan 23;20(1):e0013930. doi: 10.1371/journal.pntd.0013930 (PMC12854452; doi:10.1371/journal.pntd.0013930)
Supplement: Attachment — Submitted filename: Responses to Reviewers.pdf [file pntd.0013930.s003.pdf]

Responses to Editors and Reviewers

Additional Editor Comments

Your manuscript has been reviewed by three experts. Each reviewer considers this a valuable contribution but also has provided some suggestions for clarification and improvement. Please evaluate these comments, modify your manuscript as you think and provide a summary of how you responded to reviewer comments.

Response: Dear Editors, we are appreciative of your timely progress in our manuscript review process. We critically viewed the reviewers' comments, questions, and suggestions. We saw all of them as invaluable to improve our manuscript. Below, we provided point-by-point responses to reviewers' comments.

Dear reviewers (reviewer #1, reviewer #2, and reviewer #3), we are appreciate all of you for devoting your time in reviewing our manuscript. All of you raised important comments and added your suggestions that could help us improve our work. Below, we have provided point-by-point responses to your comments that you raised in each section of our manuscript.

Reviewers' comments:

Reviewers' Responses to Questions

Key Review Criteria Required for Acceptance?

As you describe the new analyses required for acceptance, please consider the followings:

Methods

-Are the objectives of the study clearly articulated with a clear testable hypothesis stated?

-Is the study design appropriate to address the stated objectives?

-Is the population clearly described and appropriate for the hypothesis being tested?

-Is the sample size sufficient to ensure adequate power to address the hypothesis being tested?

-Were correct statistical analyses used to support conclusions?

-Are there concerns about ethical or regulatory requirements being met?

Reviewer #1:

1. The general objective is stated, but no explicit or testable hypothesis is formulated, and the aims could be more clearly defined.

Response: Thank you for your insightful suggestion. As a result, we have revised our objectives and developed testable hypotheses. We postulated that "the pooled levels of knowledge, favourable attitude, and appropriate practices regarding anthrax prevention and control in Ethiopia vary significantly across populations, study years, and regions," despite

the fact that systematic reviews and meta-analyses do not normally test causal hypotheses. The research questions section (page 6, lines 160–163) now includes this adjustment.

2. A systematic review and meta-analysis is appropriate for synthesizing KAP evidence, although methodological reporting needs clarification.

Response: We are appreciative of your constructive comments. We carefully evaluated our methods part and attempted to improve its clarification. Specifically, we have clarified the search strategy, explicitly described eligibility criteria, and aligned methodological reporting with PRISMA 2020 guidelines. These revisions have been incorporated in the methods section (Pages 5-8, Lines 134-237).

3. The population is described, but the included groups (community, livestock owners, and professionals) are heterogeneous, which complicates interpretation.

Response: We appreciate your feedback. We agree that the included population groups are heterogeneous. The multisectoral nature of anthrax prevention and control in a One Health context is reflected in this variability. We specifically addressed population heterogeneity as a constraint that could impact the interpretation of pooled data and performed subgroup analyses. Actually, the data show that KAP levels vary little amongst population groupings, which are now shown individually in the results section. Please refer to pages 16-17, lines 328-343.

4. The total sample across studies is large.

Response: We thank the reviewer for this observation. The large cumulative sample size strengthens the precision and robustness of the pooled estimates.

Reviewer #2:

1. The authors conducted a systematic review and meta-analysis. The review protocol was registered in PROSPERO.

Response: We thank the reviewer for noting this. The protocol registration in PROSPERO was intended to enhance transparency and methodological rigor.

2. The objective of the review could be improved.

Response: We are appreciative of your helpful suggestion. As a result, we have revised the objective to be more precise and in line with the review questions. The Search strategy and review question section (page 6, lines, 153-166) now more clearly states the revised objective.

3. The search strategy could be improved. It is not clear and a justification is needed as to why the year for search strategy was not stated.

Response: Thank you for this comment. In order to make the search approach more clear and to clearly express and justify the time frame employed, we have revised the Methods section. To guarantee thorough identification of all relevant KAP studies, our search was not limited by publication year-no study/publication year's restrictions.

4. Also only 17 studies were eligible from 2016 to 2025. Any explanation as to why no studies were eligible before 2016.

Response: Thank you for this observation. Although there was no restriction to year of publication, no studies were accessed before 2016 that fulfilled the inclusion requirements. This could be because there weren't many anthrax-specific KAP studies with quantifiable data that could be extracted prior to that time.

Reviewer #3:

1. Yes

Response: Thank you. We appreciate the confirmation and have noted this.

Results

-Does the analysis presented match the analysis plan?

-Are the results clearly and completely presented?

-Are the figures (Tables, Images) of sufficient quality for clarity?

Reviewer #1:

1. The main planned analyses (random-effects meta-analysis, subgroup analyses, meta-regression, and publication bias assessment) are performed, but some analytical steps-such as harmonizing KAP definitions-are not clearly aligned with the stated plan.

Response: We are appreciative of your insightful suggestion. In order to assure comparability among included studies, we have revised the methods section and have described how diverse KAP definitions and scoring systems were harmonized into common categories using predetermined criteria. The outcome definitions and measurement section now includes this adjustment (page 7, lines 202-205).

2. The results are detailed and comprehensive, but high heterogeneity is insufficiently interpreted, and some sections (e.g. One Health findings, local disease names) are overly descriptive and not essential to the core results.

Response: We thank the reviewer for this insightful comment. We concur that there should be cautious when interpreting substantial heterogeneity. By demonstrating how results from subgroup analyses and meta-regression are connected to observed heterogeneity, we clearly emphasised the interpretation of heterogeneity in the discussion. We have also tried to minimise results that are overstated.

3. The tables and forest plots are informative.

Response: Thank you for this comment. We are pleased that the tables and forest plots clearly convey the findings.

Reviewer #2:

1. Yes, the results match the analysis plan and the results are well presented and explained.

Response: We thank the reviewer for this positive and encouraging comment.

2. Figures and tables are appropriate.

Response: We are appreciative of your detailed view.

3. However, the abstract requires improvement to include the reference for the protocol registration, search strategy with key terms. Include inclusion and exclusion criteria.

Response: We thank the reviewer for this constructive and insightful suggestion. The abstract has been revised to include the PROSPERO registration reference, key search terms, and main inclusion and exclusion criteria.

Reviewer #3

1. Yes

Response: We are appreciative of devoting your time in reviewing our manuscript.

Conclusion

-Are the conclusions supported by the data presented?

-Are the limitations of analysis clearly described?

-Do the authors discuss how these data can be helpful to advance our understanding of the topic under study?

-Is public health relevance addressed?

Reviewer #1:

1. The main conclusions align with the overall findings.

Response: We thank the reviewer for noting this.

2. Some limitations are acknowledged, but major issues such as inconsistent KAP definitions, regional imbalance of studies, and extreme heterogeneity require stronger emphasis.

Response: We thank the reviewer for this invaluable comment. We have revised the limitations section to more strongly emphasize inconsistent KAP definitions, regional imbalance of studies, and high heterogeneity, and to discuss their implications for interpretation and generalizability.

3. The discussion highlights the need for One Health approaches and improved community awareness, though the link between findings and actionable insights could be more specific.

Response: Thank you for raising this valuable comment. We have revised the discussion to more explicitly link the findings to actionable One Health-oriented recommendations and policy relevant actions.

4. The manuscript clearly outlines public health implications, particularly regarding anthrax transmission, outbreak preparedness, and One Health policy needs.

Response: Thank you for this comment. We are pleased that the public health implications and One Health policy relevance are clearly articulated.

Reviewer #2:

1. Conclusions presented reflect the results of data analysis.

Response: Thank you for giving this encouraging comment.

2. Limitations presented well. Further limitation to explain why only a few studies were conducted in a few regions in Ethiopia.

Response: We agree with the reviewer and have revised the limitation section to clarify that the concentration of studies in a few regions may reflect weak disease surveillance system, limited publication of regional data, and possible publication bias. Regions with few studies may still experience substantial disease burden that remains undocumented in the published literatures.

3. General write up needs improvement in terms of grammar and statements like line 73-74; 88-89 lacking references, line 287, 384 with unclear statements.

Response: We thank the reviewer for this important comment. The manuscript has been carefully revised to improve overall grammar, clarity, and consistency. Statements in lines

73-74 and 88-89 have been revised and supported with appropriate references. Additionally, unclear statements in lines 287 and 384 have been rephrased to improve clarity and precision. (See line 296 and 391 in revised manuscript).

Reviewer #3:

1. Yes

Response: We thank the reviewer for investing time in reviewing our manuscript.

Editorial and Data Presentation Modifications

Use this section for editorial suggestions as well as relatively minor modifications of existing data that would enhance clarity. If the only modifications needed are minor and/or editorial, you may wish to recommend "Minor Revision" or "Accept".

Reviewer #1:

1. Several sections-particularly the introduction and Discussion-contain redundant descriptions of anthrax epidemiology and general background information. Streamlining these sections would improve focus.

Response: We are appreciative of your careful observation and giving insightful suggestions. We have carefully revised our manuscript and redundant descriptions have been streamlined accordingly.

2. PRISMA guidelines require transparent reporting of the complete search strings for each database. These should be added as supplementary material.

Response: We thank the reviewer for highlighting this point. To ensure full compliance with PRISMA guidelines, we have included the complete search strings for each database as a supplementary material.

3. The reported search period (August-September 2025) and the inclusion of studies up to 2025 require verification and correction.

Response: Thank you for this important observation. We confirmed that the literature search was conducted between August and September 2025, with the final search completed on 15 September 2025. The manuscript has been revised to accurately reflect this and to clarify the inclusion of studies published up to that date.

4. Clarify reporting of grey literature: specify whether unpublished studies were identified, assessed, or excluded.

Response: We thank the reviewer for this important comment. Grey literature sources were searched, but the full texts could not be accessed; therefore, unpublished studies were not assessed and or included. We have indicated this clarification in our revised manuscript in methods section (page 7, lines 183-185) and results section (page 9, lines 255-258).

5. Strengthen and the limitations: emphasize the high heterogeneity, variability in outcome definitions, and regional imbalance of included studies.

Response: We thank the review for highlighting this important suggestion. We have carefully revised our manuscript and tried to improve all issues you suggested.

Reviewer #2:

1. Minor as indicated in the previous sections

Response: We are appreciative of your comments. The minor issues noted have been addressed as indicated in the previous responses.

Reviewer #3:

1. No

Response: We appreciate the reviewer's feedback. We note that minor revisions alone may not be sufficient, and we have revised the manuscript thoroughly to improve clarity, accuracy, and completeness.

Summary and General Comments

Use this section to provide overall comments, discuss strengths/weaknesses of the study, novelty, significance, general execution and scholarship. You may also include additional comments for the author, including concerns about dual publication, research ethics, or publication ethics. If requesting major revision, please articulate the new experiments that are needed.

Reviewer #1:

1. This manuscript addresses an important and timely public health issue in Ethiopia by synthesizing national-level evidence on community knowledge, attitudes, and practices (KAP) related to anthrax prevention and control. The topic is highly relevant to One Health policy implementation. The authors present the first pooled national estimates on KAP outcomes for anthrax, which represents a notable contribution to the existing literature.

Response: We thank the reviewer for the encouraging remarks and for recognizing the relevance and contribution of our study to One Health policy and public health interventions in Ethiopia.

2. Strength of the study include: clear public health and One Health relevance; a comprehensive search across multiple databases; use of established frameworks (PRISMA JBI, CoCoPop); application of appropriate statistical approaches such as random-effects meta-analysis, subgroup analysis, publication bias assessment, and sensitivity testing; inclusion of detailed contextual information that highlights real-world risk factors and behaviours contributing to anthrax transmission.

Response: We thank the reviewer for the positive comments and for recognizing the strengths of our study, including its relevance, methodology, and analytical rigor.

3. However, several weaknesses limit the clarity and interpretability of the findings: Definitions of "good knowledge," "positive attitude," and "good practice" are not standardized across primary studies, leading to inconsistent outcome measurement;

Response: We thank the reviewer for this insightful comment. As we responded above, we have revised our manuscript and have indicated that the definitions of KAP outcomes have been harmonized to be consistent in data extraction, analysis, and results interpretations.

4. The introduction and discussion contain redundancies and could be made more concise and focused:

Response: Thank you for this comment. We have carefully revised our manuscript to be improved accordingly.

5. The implications for One Health policy, although noted, would benefit from more actionable and specific recommendations.

Response: We thank the reviewer for this observation. As we responded above, we have revised it.

6. Overall, the manuscript is well-conceived and addresses a significant gap in the field. The revisions required are primarily editorial and related to improved transparency, clarification of methodology, and stronger discussion of limitations rather than new analyses or additional data collection.

Response: We appreciate the reviewer for raising this thoughtful comment. We have carefully revised all sections of our manuscript and have tried to address the issues you suggested including grammatical and sentence structure issues, methodological rigors and clarifications, and limitations.

Reviewer #2

1. Abstract requires improvement and clarity for search strategies should be provided. Include in the methods sections how different databases were search based on Boolean Operators.

Response: We appreciate your suggestions. We have revised the abstract and have improved accordingly, and in methods section we have indicated that search strings for each database have been provided as supplementary material.

2. Check for grammar and improper statements

Response: Thank you for observation. We have carefully checked and amended it.

Reviewer #3

1. The authors reviewed and analyzed previous studies on public health issues related to anthrax infections in Ethiopia. These studies covered transmission routes and residents' understanding of infection control measures. While information covering the entire country is unavailable, no similar meta-analysis has been conducted to date. This report contains valuable information for developing anthrax control measures in Ethiopia.

Response: We thank the reviewer for giving comments and recognized our study as relevant to the prevention and control of anthrax in Ethiopia.
